# Comparison of Complications in Percutaneous Dilatational Tracheostomy versus Surgical Tracheostomy

**DOI:** 10.5539/gjhs.v6n4p221

**Published:** 2014-04-20

**Authors:** Siamak Yaghoobi, Hamid Kayalha, Raziyeh Ghafouri, Zohreh Yazdi, Marzieh Beigom Khezri

**Affiliations:** 1 Department of Anesthesiology, Qazvin University of Medical Sciences, Qazvin, Iran; 2 School of Medicine, Qazvin University of Medical Sciences, Qazvin, Iran

**Keywords:** tracheostomy, percutaneous, dilatational tracheostomy, blue rhino

## Abstract

**Background:**

Tracheostomy facilitates respiratory care and the process of weaning from mechanical ventilatory support.

**Aims:**

To compare the complications found in percutaneous dilatational tracheostomy (PDT) and surgical tracheostomy (ST) techniques.

**Methods:**

This was a prospective randomized study to evaluate the complications of PDT and ST procedures in patients admitted to ICU unit of a teaching hospital during 2008 to 2011.

We studied 40 patients in each group. PDTs were performed with blue rhino technique at the bedside by a skilled clinician and all cases of STs performed by Charles G Durbin technique in operating room under general anesthesia. Bronchoscopic examination through tracheostomy tube was performed to ensure the correct position of tracheostomy tube in the trachea lumen. The duration of procedures and pre- and post-interventional complications were recorded.

**Results:**

The most common complications observed in the PDT group were minor bleeding (n=4), hypoxemia, and cardiac dysrhythmias (n=3) whereas in the ST group, the most frequent complications were minor bleeding (n=5) and endotracheal tube puncture (n=3). The difference in overall complications between the two groups was insignificant (P=0.12).

**Conclusion:**

PDT with blue rhino technique is a safe, quick, and effective method while the overall complications in both groups were comparable.

## 1. Introduction

Tracheostomy is considered as the airway management of choice for patients who need prolonged mechanical ventilation support or airway protection (Boonsarngsuk, Kiatboonsri, & Choothakan, 2007). It facilitates the respiratory care and the process of weaning from mechanical ventilatory support (Rudr, 2002).

Tracheostomy has several advantages over translaryngeal intubation including better patient tolerance, reduced laryngeal irritation, easier nursing care, enhanced ability to communicate, and reduced dead space and work of breathing (Griffiths, Barber, Morgan, & Young, 2005). Approximately 5 to 13% of patients on mechanical ventilation will be in need of prolonged mechanical ventilation (>21 days). In these patients, the critical care personnel have to make a decision on whether and when to perform a tracheostomy (Bartolome, 2012). This decision is individually based on the risk and benefits of tracheostomy and prolonged intubation and also the patient’s family preferences and expected clinical outcomes. Nowadays, most intensivists agree that if a patient is in need of MV for more than 10–14 days, a tracheostomy is indicated and should be planned under optimal conditions (Bartolome, 2012). However, tracheostomy can be performed at intensive care units (ICU) as a bed side procedure or in an operating room setting (Durbin, 2005). With advanced technology and increasing interest in minimally invasive procedures, variations on standard open surgical tracheostomy have been evolved over the recent years. Therefore, it seems that percutaneous dilatational tracheostomy has gained the popularity to become a common method in critically ill patients. Dilatational tracheostomy might be performed through several different approaches and systems. In ICUs, the most common indication for the application of PDT is a need for prolonged mechanical ventilation in cases such as severe brain injury, acute respiratory distress syndrome, and severe chronic obstructive pulmonary disease or multiple organ failure. However, indications, risks, benefits, timing, and the technique of the procedure remain controversial and depend on the clinical condition of the patient in particular the respiratory status. (Durbin, 2005; Groves et al., 2007). Furthermore, the ability of clinicians to predict which patients required extended ventilatory support was limited (Young et al., 2013). (Young, Harrison, Cuthbertson, Rowan; TracMan Collaborators, 2013).

It is reported that tracheostomies with blue rhino technique is a modified version of the Ciaglia technique (Ciaglia, Firsching, Syniec,1985; Byhahn et al., 2000), in which the dilatation of the stoma is performed in a single step by means of a hydrophylically coated, curved dilator- the blue rhino. Therefore, insertion of tracheostomy tube was performed more rapid and the risk of posterior tracheal wall injury and intraoperative bleeding was reduced while the adverse effect on oxygenation during repeated airway obstruction by the dilators was avoided.

In Iran, PDT is performed only in limited number of centers and there is little data about the outcome. The aim of the present study was to compare the surgical tracheostomy with PDT in intensive care patients, with special consideration on technical difficulties including the duration of procedure, and the intra- and post-operative complications.

## 2. Methods

### 2.1 Design and Participants

This was a randomized prospective analysis to compare the results of PDT with surgical tracheostomy performed on patients admitted to ICU unit of a teaching hospital from March 2008 to September 2011. Inclusion criteria were all critically ill patients in the general ICUs with age>18, the necessity of mechanical ventilation for ≥2 week, and hemodynamic stability. Exclusion criteria included infection of tracheostomy site, abnormal neck anatomy, known or suspected difficult endotracheal intubation, unstable cervical spine, age <18 years, uncorrectable coagulopathy, hemodynamic instability, and a history of previous tracheostomy. Informed written consents were obtained from the relatives and the study was prospectively approved by the Hospital’s Ethics Committee for human studies. All patients were randomized into two groups using computer-generated random numbers.

### 2.2 Tracheostomy Procedures

All PDTs were performed with blue rhino technique kit at the bedside in the ICU and all surgical tracheostomies in the operating room, both under general intravenous anesthesia. The drugs used for the induction of anesthesia consisted of fentanyl (50-100 µg), midazolam (2 mg), propofol (1-2 mg/kg), and atracurium (0.5 mg/kg), prescribed intravenously. This protocol also included continuous intravenous propofol (50 mg/kg/min). All patients were preoxygenated with 100% oxygen for 8-10 minutes and the vital signs, pulse oximetry, and electrocardiograms were continuously monitored throughout. All PDTs were performed by the same intensivist and all surgical tracheostomies by the same surgeon. The tracheostomy site was marked 3 cm above the sternal notch. Lidocaine 1% with 1/200,000 diluted epinephrine was infiltrated subcutaneously to reduce bleeding. Later, the fiberoptic bronchoscope was placed in the endotracheal tube and in case of the presence of endotracheal secretions it was removed through suctioning. While the cuff of the tracheal tube was deflated and the rings of trachea were visualized with bronchoscope to prevent puncture, the tube was withdrawn until the tip was just in the larynx. Over the guidewire and guiding catheter, the blue rhino was passed to the appropriate skin level marking, resulting in tracheal dilation. Finally, the tracheostomy tube was inserted through the tracheal stoma. Later, the introducer, the guidewire, and the guiding catheter were removed, leaving the tracheostomy tube in situ. The cuff of the tracheostomy tube was fully inflated, the ventilator breathing circuit connected, and the tube was fixed with tapes around the neck. Satisfactory ventilation was verified bilaterally by auscultation of the chest. Tracheal suctioning was performed to remove secretions and blood. Surgical tracheostomies were performed by Charles G Durbin technique (Durbin, 2005).

### 2.3 Measures

The duration of procedures and pre- and post-interventional complications including hypotension, hypoxemia, cardiac dysrhythmias, difficult tube placement, endotracheal tube cuff leak, minor and major bleeding, tracheostomy-associated death, stomal infection, posterior tracheal wall injury, and tracheal ring fracture were recorded and compared. The presence of surgical emphysema at the site was also observed and chest X-ray performed to check for tube position and pneumothorax.

### 2.4 Statistical Analysis

This study was powered on the basis of previous results showing an incidence rate of 30% for complications in the surgical tracheostomy (Ben-Num, Altman, & Best, 2004). A sample size of 40 patients in each group was calculated to detect a decrease in the incidence of complications down to 15% with α=0.05 and β=0.2. The chi-square test or Fisher’s exact test was applied to determine the differences in the occurrence of complications between the groups. For continuous variables, student’s t-test was applied to compare the data between the two groups. Differences were considered to be statistically significant if p<0.05.

## 3. Results

As shown in Table 1, a total of 40 patients were considered for PDT. In the PDT group, 22 cases were men and 18 women (M/F=22/18), with a mean age of 35±11.8 years. Also, 40 patients were considered for surgical tracheostomy including 19 men and 21 women (M/F=19/21) with a mean age equal to 32±12.6. There was no difference between the two groups in terms of age and sex (p>0.05). The procedure time as defined by the time span from the first puncture of the trachea to the end of successful insertion of the tracheostomy tube and connection to ventilator for PDT and ST groups was 10.01±2.42 minutes and 15.08±3.16 minutes, respectively. All PDTs were performed without technical difficulties with the exception of one case in whom the procedure was converted to surgical tracheostomy due to technical difficulty. There was no significant disturbance of vital signs throughout the procedures. There was no death related to tracheostomy. Also, there was no stomal infection in patients of the present study. Posterior tracheal wall injury and major bleeding were only observed in one patient of PDT group and one case of ST group, respectively. Other complications such as tracheal ring fracture and pneumothorax or death because of procedure were not observed in the patients of two groups. Table 2 shows the comparison made between the post procedure outcomes for the PDT and surgical groups.

**Table 1:** Demographic data and duration of procedure time for two study groups

Groups	PDT (n =40)	ST (n =40)	P value
Age (years)	35.21±11.81	32.08±12.63	0.09
Gender(F/M)	18/22	21/19	0.12
Duration of tracheostomy (min)	10.01±2.42	15.08±3.16	<0.001

Data are presented as mean±SD or number of patients. PDT = percutaneous dilatational tracheostomy, ST = surgical tracheostomy.

**Table 2:** Postoperative complications of blue rhino PDT and surgical tracheostomy

Complications	Pdt	St	P Value
No complication	25	27	0.09
Complication	15	13	0.12
Hypotension	2	1	0.08
Hypoxemia	2	0	<0.001
Tracheal ring fracture	0	0	-
Endotracheal tube puncture	0	3	<0.001
Cuff leak of tube	0	1	<0.001
Difficult tube placement	1	0	<0.001
Stomal infection	0	0	-
Minor bleeding	4	5	0.21
Major bleeding	0	1	<0.001
Death related to tracheostomy	0	0	-
Pneumothorax	0	0	-
Posterior wall injury	1	0	<0.001
Subcutaneous emphysema	1	1	-
Cardiac dysrhythmias	3	1	<0.001
False passage	1	0	<0.001

Data are presented as number. PDT= percutaneous dilatational tracheostomy, ST=surgical tracheostomy.

## 4. Discussion

Based on the data found in our study, it was concluded that PDT with blue rhino technique is a safe, quick, and effective method and can be performed by an intensivist while the overall complication rate in both group was comparable. In our study, the overall complication rate was 40% and the majority of complications were minor and quickly improved. This was in contrast with the study by Siranovic and coworkers in which a complication rate of 22.5% was reported (Cattano, Giunta, & Buzzigoli, 2006; Širanović et al., 2007). Meanwhile, despite the long experience with ST, the overall complication rate of ST is still high. In surgical tracheostomy, the incidence of local hemorrhage or stomal infection was reported to be around 37% (Cattano et al., 2006). However this discrepancy between the results may be due to different population, physicians’ skill, and approach to procedures. The mean procedure time of 10 minutes found in this study was similar to those demonstrated in previous reports (Hinerman, Alvarez, Keller, 2000; Freeman, Isabella, Lin, & Buchman, 2000). On average, the time spent in performing PDT was approximately 5 minutes less than ST. This resulted in a shorter duration and a lesser chance of hypoxia during the procedure. This finding is consistence with the results of the study by Siranovic et al in which PDT technique with Griggs method was associated with a shorter procedure time and lower morbidity in comparison to the surgical technique (Širanović et al., 2007). Previous studies indicate that early PDT-related complications include paratracheal insertion, posterior tracheal wall injury, major bleeding, and pneumothorax (Freeman et al., 2000; Dongelmans et al., 2004; Tomsic et al., 2006). These complications, however, were not found in our study. This might be due to the application of bronchoscopic guidance which provided the operator with direct visual information, assuring the correct placement of tracheostomy and avoiding possible complications and this is the main reason for recommending the use of bronchoscopic guidance as a part attached to PDT by many authors (Oberwalder et al., 2004; Tomsic et al., 2006; Petros et al., 1997).

In agreement to our study, Cantais, Kaiser, Le-Goff and Palmier (2002) also demonstrated that PDT is a safe procedure to perform at the bedside. In general, PDT appears to be a less traumatic procedure and the skin incision required in PDT is smaller than in ST. Furthermore, PDT requires tracheal opening (tracheostoma) by dilation of the soft tissue space between the tracheal rings instead of direct cutting through cartilaginous ring as in ST. Therefore, a lower incidence of tracheal stenosis at stomal site would be expected. Several studies have shown the significant cost saving in western countries, however, the main limitation of PDT in Iran is the high cost of the commercial kit.

## 5. Conclusion

In summary, our data suggest that PDT with blue rhino technique is a safe, quick, and effective method and can be performed by an intensivist. Moreover, the overall complication rate in both groups was comparable, although the procedure time was shorter in PDT. The main advantage of PDT is the possibility of its performance in ICU, as a bedside procedure, which prevents the risk of transfer to the operating room. However, it is clear that the simplicity and easiness of a technique shouldn’t lead to an attitude that every physician is allowed to perform it. PDT must be left in the hands of physicians with enough experience. Since there is some concern that the evaluation of morbidity and outcomes of patients with a tracheostomy has not, at present, been adequately investigated, further multi-center, large scale trials are needed to achieve a better conclusion.
